# Quantitative Criteria for Solvent Selection in Liquid-Phase Exfoliation: Balancing Exfoliation and Stabilization Efficiency

**DOI:** 10.3390/nano15050370

**Published:** 2025-02-27

**Authors:** Shunnian Wu, W. P. Cathie Lee, Hashan N. Thenuwara, Ping Wu

**Affiliations:** Entropic Interface Group, Engineering Product Development, Singapore University of Technology and Design, 8 Somapah Road, Singapore 487372, Singapore; shunnian_wu@sutd.edu.sg (S.W.); cathie_lee@sutd.edu.sg (W.P.C.L.); hashan_thenuwara@mymail.sutd.edu.sg (H.N.T.)

**Keywords:** liquid-phase exfoliation, binding energy, exfoliation energy, solvents, dipole moment, planarity, polarity, biaxial straining

## Abstract

The selection of solvent is pivotal in liquid-phase exfoliation (LPE), as an ideal solvent minimizes the energy required to disrupt the interlayer attractive interactions while stabilizing exfoliated layers to prevent re-agglomeration. This study theoretically analyzed the LPE of Mg(OH)_2_ in different solvents, including water, dimethyl sulfoxide (DMSO), dimethylformamide, N-methyl-2-pyrrolidone (NMP), isopropyl alcohol, and 2-butanone, through first-principles calculations combined with experimental validation. DMSO was identified as the most effective solvent for reducing the interlayer attraction, based on exfoliation energy calculations, while NMP was the most efficient for stabilizing exfoliated layers, based on binding energy assessments. Principal component analysis of the solvents’ physicochemical properties reduced the original dataset of seven variables to two dominant factors. The binding energy correlates with planarity and polarity, whereas the exfoliation energy is governed by dipole moment and polarity. The biaxial straining theory successfully clarified the underlying mechanisms behind the established criteria for selecting the optimal solvent. Experimental results confirmed that DMSO outperforms water in the LPE of Mg(OH)_2_. These results establish a quantitative framework for solvent selection, enhancing the efficiency of the LPE processes.

## 1. Introduction

Two-dimensional (2D) nanomaterials are attracting increasing interest due to their exceptional mechanical, optical, and electrical properties [1,2]. Common preparation techniques include top–down and bottom–up approaches [3,4]. Bottom–up techniques involve constructing nanoscale materials from atomic or molecular precursors and include methods such as molecular beam epitaxy, wet chemical method, sol–gel method, hydro/solvothermal synthesis, template synthesis, microwave-assisted method, topochemical transformation, chemical vapor deposition (CVD), and physical vapor deposition (PVD). The top–down technique, on the other hand, carves nanoscale structures by carefully removing components from larger or bulk objects. This approach includes methods such as electrochemical exfoliation, micromechanical exfoliation, ultrasonic exfoliation, liquid-phase exfoliation (LPE), lithium-intercalation exfoliation, ion-change exfoliation, laser ablation, and sputtering. The LPE method has stood out among these methods recently due to its affordability and high efficacy [5,6,7].

Liquid-phase exfoliation is based on the principle that transforming a layered crystal into nanosheets within a liquid medium involves two critical steps: first, breaking the interlayer bonds to detach the nanosheets from the bulk crystal, and second, stabilizing the exfoliated nanosheets to prevent their reaggregation. This method was originally developed to produce large quantities of graphene back in 2008 [8,9]. The efficiency of LPE for graphene production is influenced by the interaction between the solvent and graphite layers, which leads to the unzipping and peeling off of thin graphite layers [10]. Solvents are required to have a certain surface energy in order to overcome the van der Waals forces, and a surface tension (γ) of approximately 41 mJ m^−2^ is necessary to provide the energy required to break the bonds between layers [8,11]. Commonly used solvents for liquid-phase exfoliation (LPE) of graphene include N-methyl-2-pyrrolidone (NMP), N,N′-dimethylformamide (DMF), and ortho-dichlorobenzene (o-DCB). This method has also been successfully applied to exfoliate a wide range of two-dimensional (2D) materials beyond graphene, such as phosphorene, boron, WO_3_, MoO_3_, LiMn_2_O_4_, and FeS_2_ [5].

The choice of solvent is a critical determinant in the exfoliation process, and it is required to fulfil three essential criteria: (1) effectively transmit the exfoliating force, (2) minimize the energy required to overcome the interlayer attractions, and (3) stabilize the exfoliated layers by preventing re-agglomeration through steric hindrance [12]. Numerous organic solvents, including dimethyl sulfoxide (DMSO), DMF, NMP, isopropyl alcohol (IPA), and 2-butanone (MEK), have been employed as exfoliation media in recent studies [5]. Additionally, co-solvent systems, like water combined with NMP, significantly affect both the yield and stability of the exfoliated nanosheets [13]. The yield and stability of the exfoliated nanosheets are generally dependent on three fundamental factors: solid–liquid interfacial energy, Hansen solubility parameters (HSP), and physical parameters sensitive to the intermolecular interactions [13]. Additionally, the Hildebrand solubility parameters have been proposed as potential criteria for solvent selection. It is suggested that a solvent’s effectiveness in both the exfoliation and dispersion of a nanomaterial is closely tied to a match between the cohesive energies of the solvent and the material being exfoliated [11]. This “like dissolves like” hypothesis attempts to correlate both the exfoliation efficiency and dispersion stability with the extent to which a solvent can “dissolve” a given nanomaterial [11]. However, there is evidence that both the exfoliation and the colloidal aggregation of nanomaterial flakes are linked to multiple energetic and structural details resulting from interactions between closely separated flakes and intercalating, confined solvent molecules [14]. Therefore, despite the diverse range of exfoliation media explored by researchers, there remains a significant knowledge gap in designing optimal solvent systems for efficiently exfoliating two-dimensional materials and stabilizing the resulting nanosheets. The selection of solvents for exfoliation still predominantly relies on trial-and-error methods rather than established quantitative indices based on the physicochemical properties of the solvents and solvent–nanomaterial interactions.

While the hydrothermal crystal growth technique has been used to produce 2D Mg(OH)_2_ [15], reports on its preparation via liquid-phase exfoliation (LPE) remain absent. Here, we adopt an integrated approach combining density functional theory (DFT) simulations and experimental validations to systematically screen the solvents. Mg(OH)_2_ serves as a model material for demonstrating this framework. DFT provides a robust tool for quantifying the interactions at the solvent–nanomaterial interface. By simulating Mg(OH)_2_ exfoliation and corroborating the results with experimental data from different solvents, we aim to identify the most effective solvent for both exfoliation and stabilization. This combined approach not only facilitates the establishment of quantitative indices for solvent selection but also provides a comprehensive understanding of the LPE process for Mg(OH)_2_ and other layered nanomaterials.

## 2. Materials and Methods

### 2.1. Computational Methods

First-principles calculations were carried out using the Vienna ab initio simulation package (VASP) [16], employing the Perdew–Burke–Ernzerhof (PBE) generalized gradient approximation (GGA) for the exchange–correlation functional [17]. A projector augmented wave (PAW) method [18,19] was used to describe the electron–core interactions within a plane-wave basis set framework. A kinetic energy cutoff of 500 eV was applied for the plane-wave expansion. To account for van der Waals interactions, the DFT + D3 correction scheme developed by Grimme et al. [20] was utilized. The convergence criterion for total energy was set as 1.0 × 10^−6^ eV, and the forces on individual atoms were minimized to be below 0.01 eV/Å for geometry optimization and total energy calculations. A smearing value of 0.01 eV was maintained throughout. The Brillouin zone was sampled using a Monkhorst–Pack [21]. The number of K-points (NK) was adjusted such that (NK × L) (where L is the lattice constant) was approximately 25 Å for structural relaxations and 45 Å for electronic structure calculations. This ensured precision in both geometry optimization and electronic property evaluations.

Each solvent molecule was placed in a 20 Å × 20 Å × 20 Å cubic unit cell and fully optimized. Their structural data are summarized in Section 1 of the SI. The Connolly surface area and volume of each solvent were calculated with Materials Studio 8.0 (Biovia, San Diego, CA, USA) [22] using their optimized structures, respectively. The Connolly surface is defined as the envelope traced out by the probe sphere (typically representing a solvent molecule) as it rolls over the van der Waals surface of the molecule. It provides a more realistic depiction of the accessible surface compared to the van der Waals surface [23,24]. We defined planarity as the Connolly surface volume divided by the Connolly surface area with the unit Å.

The previously optimized Mg(OH)_2_ crystalline structure [25] is used in this work. Its crystalline structure was cleaved in the most stable (001) direction [26] to construct the 4 × 4 × 3 slab model. A full structural optimization was performed to determine the binding energy of the Mg(OH)_2_ surface with different solvents, keeping the bottom eight layers fixed. The exfoliation energy was calculated by inserting various solvents into a 4 × 4 × 1 Mg(OH)_2_ bilayer. The surface and bilayer structure data are summarized in Section 2 and Section 3 of the SI, respectively. All the optimized structures were visualized using VESTA 3 [27].

### 2.2. Experimental Procedure

Mg(OH)_2_ powder of purity ≥ 99% (BioUltra) (particle size > 1 µm) and DMSO (ACS reagent) of purity ≥ 99.9% were purchased from Sigma Aldrich (Merck Pte. Ltd., Singapore, Singapore). All the chemicals were used as received without further purification. Neptec Halios lab water system (NEPTEC GmbH, Elbtal, Germany) was employed to generate deionized water.

Thinky nano-premixer (PR-1) ultrasonic mixer instrument (Thinky Corporation, Tokyo, Japan) was utilized for the liquid-phase exfoliation experiment. Both deionized water and DMSO were used separately as solvents for the exfoliation. An amount of 0.5 g of Mg(OH)_2_ powder was mixed with 10 mL of solvent in the nano-premixer vial, and the sample was sonicated with the following sonication profile: first 10 min for sonication, then 2 min of still period, and finally another 10 min for mixer and sonication again.

After the sonication process, the sample was centrifuged with BIOBASE high speed centrifuge (BIOBASE Group, Jinan, China) at 6000 rpm for 30 min using water as solvent. The supernatant was freeze-dried to obtain the nanoparticles, which is denoted as sample A for characterization. The sample with DMSO as solvent was washed by diluting 5 mL sonicated solution with 20 mL deionized water and subsequently centrifuging at 14,000 rpm for 10 min. The washing process was repeated 3 times to ensure no remaining solvent. Then, the washed samples were centrifuged at 6000 rpm, and the obtained supernatant was freeze-dried and is denoted as sample B for characterization.

Morphology of the samples was analyzed using Field Emission Scanning Electron Microscopy (SEM) instrument (JEOL JSM-7600F, Jeol, Tokyo, Japan), and Transmission Electron Microscopy (TEM) characterizations were performed using field-emission TEM (FEI Talos F200, Thermo Scientific, MA, USA) operated at 200 kV, with energy dispersive X-ray spectroscopy (EDS) attachment to observe nanosheets after exfoliation experiment. The two samples were dispersed in ethanol separately and ultrasonicated for 2 min. Then, the sonicated samples were drop-cast on carbon film-coated Cu grids (300 meshes) for TEM analysis.

## 3. Results and Discussion

### 3.1. Structure and Property of Solvent

The structure and properties of the solvents examined in this study are listed in Table 1. Surface tension is the property of a liquid’s surface that resists external force due to cohesive forces between molecules. It plays a crucial role in determining the interaction between a liquid and a solid. Polarity and dipole moments can be employed to describe the distribution of electrical charges within molecules. A molecule’s polarity is determined by its shape and the electronegativity differences between its atoms, leading to a dipole moment—a measure of the separation of positive and negative charges within the molecule. Planarity can influence the effective utilization of interlayer space in layered materials. It is seen that water has the largest surface tension and polarity, while NMP shows the largest planarity. Meanwhile, DMSO and NMP show a substantially large dipole moment.

### 3.2. Binding of Solvent on Mg(OH)_2_ Surface

**Figure 1:**
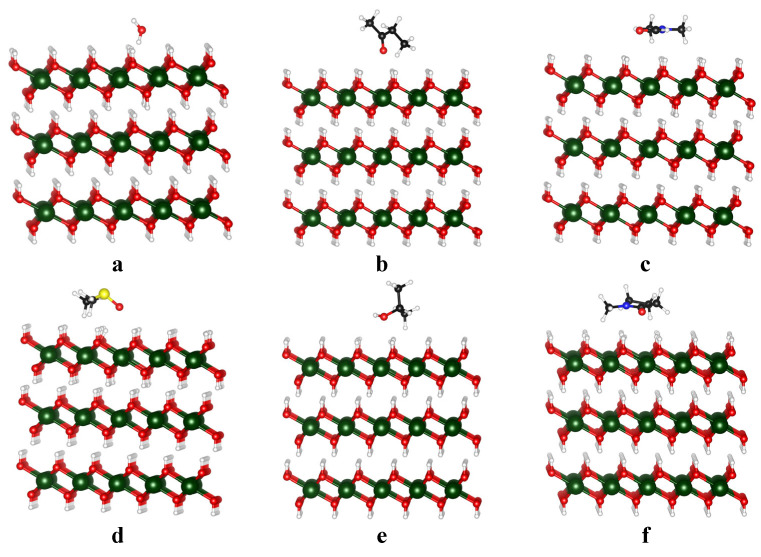
Binding of water (**a**), MEK (**b**), DMF (**c**), DMSO (**d**), IPA (**e**), and NMP to Mg(OH)_2_ surface (**f**). Black, dark green, red, blue, yellow, and white balls represent C, Mg, O, N, S, and H atoms, respectively.

The interaction between various solvents and the Mg(OH)_2_ surface was simulated to assess their capacity to stabilize the exfoliated Mg(OH)_2_ nanosheets. Stronger interactions between the solvent and the surface can effectively prevent the re-agglomeration of the nanosheets. Figure 1 illustrates the configuration of a single solvent molecule stacked on the Mg(OH)_2_ surface. Interactions between solvent molecules themselves were not considered in this work. Interestingly, DMSO, IPA, and NMP were found to align parallel to the Mg(OH)_2_ surface, whereas water, MEK, and DMF adopted a perpendicular orientation. When solvent molecules align parallel to the surface, their planar geometry can allow additional solvent molecules to stack more easily. This multilayer stacking can create a more uniform coverage, which helps in stabilizing the exfoliated layers against re-agglomeration. Despite facilitating stacking, parallel configurations may not always maximize the interaction strength with the surface. For instance, a planar orientation may limit the degree of overlap between the solvent molecule’s functional groups and the active sites on the Mg(OH)_2_ surface. This is evident in the case of IPA, where the binding energy is relatively weak (−0.37 eV) despite its parallel alignment. A perpendicular orientation introduces steric hindrance, which can restrict the stacking of additional solvent molecules on the surface. This reduced molecular stacking may negatively impact the overall stabilization of the exfoliated nanosheets. Solvents in a perpendicular orientation may exhibit stronger localized interactions with the surface. For example, DMF, which aligns perpendicularly, shows a stronger binding energy (−0.52 eV) compared to IPA. This suggests that the perpendicular configuration allows the functional groups of the solvent to interact more directly with the surface’s active sites, increasing stabilization at the molecular level. The binding energies of the studied solvents are summarized in Table 2. The binding energy is a critical indicator of the solvent’s capacity to stabilize the surface. A more negative binding energy value indicates stronger attractive interactions between the solvent and the exfoliated Mg(OH)_2_ surface, which helps prevent the re-agglomeration of exfoliated layers for stabilization. Among the solvents studied, NMP exhibits the strongest interaction with the Mg(OH)_2_ surface, whereas MEK and IPA demonstrate the weakest interactions. These significant differences underscore the importance of solvent choice in achieving effective stabilization of the exfoliated Mg(OH)_2_ nanosheets. However, molecular alignment alone does not guarantee effective stabilization. While parallel configurations are advantageous for achieving multilayer stacking, perpendicular orientations can enhance specific solvent–surface interactions. Selecting an optimal solvent for LPE requires considering both the molecular configuration and the associated binding energy to maximize stabilization while minimizing re-agglomeration. The chemical nature of the solvent, such as its functional groups and polarity, may also play an important role.

### 3.3. Insertion of Solvent in Mg(OH)_2_ Bilayers

**Figure 2:**
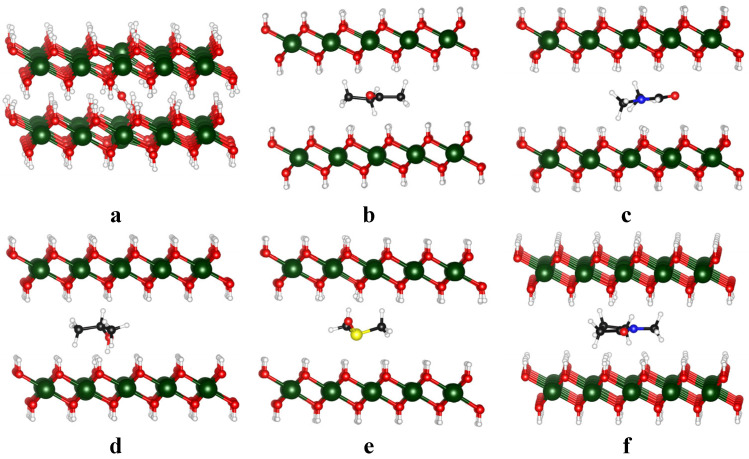
Intercalation of water (**a**), MEK (**b**), DMF (**c**), DMSO (**d**), IPA (**e**), and NMP in Mg(OH)_2_ interlayers (**f**). Black, dark green, red, blue, yellow, and white balls represent C, Mg, O, N, S, and H atoms, respectively.

Figure 2 depicts the intercalation of solvents into the bilayers of Mg(OH)_2_. The attractive energy between the bilayers can quantitatively describe the energy required to exfoliate Mg(OH)_2_. It is seen that the solvent molecules, except water, lie parallel between the bilayers. The parallel configuration is also observed when a long-chain organo-ammonium cation is inserted into the interlayers [30]. The water molecule orients perpendicularly between the bilayers, with its two hydrogen atoms forming weak covalent bonds with oxygen atoms from different layers. The distances between the two hydrogen atoms of H_2_O and the nearest neighboring oxygen atoms in Mg(OH)_2_ are 1.39 Å and 1.41 Å, respectively. These values are slightly greater than the H–O bond lengths in H_2_O, which measure 1.05 Å and 1.06 Å. However, they remain significantly shorter than the strong and mostly covalent hydrogen bonds, which range from 2.2 to 2.5 Å [31]. These weak covalent bonds impede the reduction of interlayer interactions, resulting in a slight increase in the layer spacing ($D_{i}$) from 2.50 Å to 2.77 Å upon water intercalation. In contrast, other solvents significantly increase the interlayer distance ($D_{i}$) to over 6 Å, attributed to their parallel configuration between the bilayers. This uniform parallel alignment results in slight variations in the interlayer spacing, ranging from 6.03 Å for MEK to 6.26 Å for DMSO. These distances exceed those reported for branched organo-ammonium cations and are comparable to those induced by long-chain organo-ammonium cations [30], indicating substantial separation between the bilayers. Such a large interlayer distance suggests the separation of the bilayers.

This observation aligns with the calculated exfoliation energy ($E_{ex}$), which ranges from −1.10 eV for DMSO to −1.47 for NMP, as presented in Table 2. A more negative $E_{ex}$ corresponds to stronger interlayer interactions and higher energy requirements for exfoliation. Among the solvents studied, DMSO demonstrates the greatest efficiency in weakening the interlayer attractions, as evidenced by its relatively less negative exfoliation energy. These findings highlight DMSO’s superior ability to facilitate the exfoliation process by reducing the interlayer binding forces.

### 3.4. Quantitative Structure–Property Relationship (QSPR) Analysis

**Figure 3:**
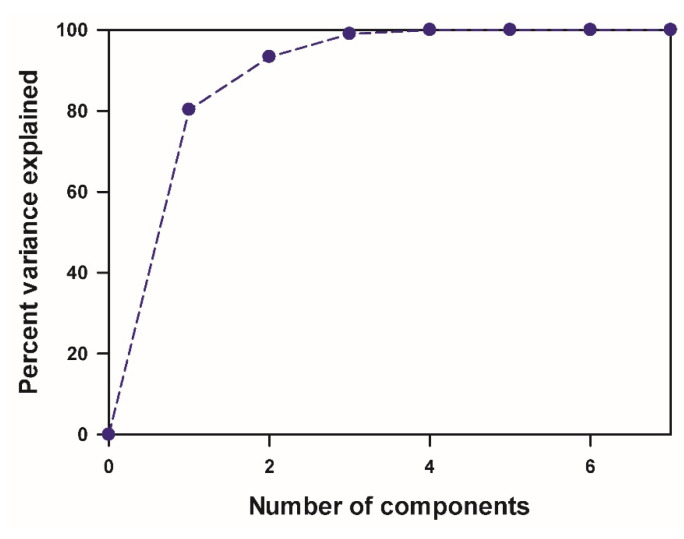
Percent variance explained by principal components.

A matrix was constructed that includes the four properties listed in Table 1, along with the Connolly surface area, volume, and interlayer spacing from Table 2. Principal component analysis (PCA) was then applied to reduce its dimensionality, facilitating the establishment of a quantitative structure–property relationship. PCA offers the advantage of condensing the number of variables while preserving most of the original information. Figure 3 illustrates the percent variance explained by the principal components. The first principal component explains 80.28% of the original data variance. Principal components 2, 3, and 4 explain 13.08%, 5.68%, and 0.96%, respectively. This indicates that two components nearly explain the original data.

**Figure 4:**
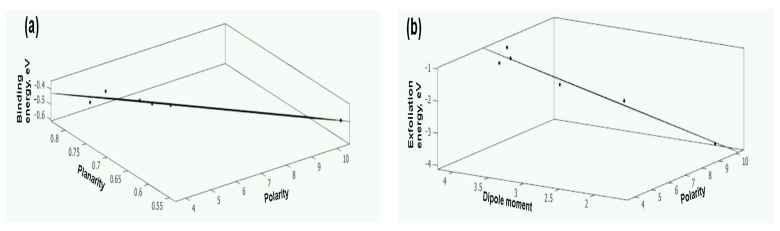
Variation in binding energy (**a**) and exfoliation energy (**b**) with the selected physical properties of solvent.

Since PCA suggests that two properties from the matrix can represent the seven properties, a quantitative structure–property relationship (QSPR) analysis was performed to identify the two key physicochemical properties influencing binding energy $\left(E_{b}\right)$ and exfoliation energy $\left(E_{ex}\right)$. Multiple regression analysis was performed between two of the seven parameters and binding energy $\left(E_{b}\right)$ and exfoliation energy $\left(E_{ex}\right)$, respectively, to establish their correlations. The equations with the highest correlation coefficients were selected. Figure 4 illustrates the trends of these energies with respect to the selected solvent properties. It is observed that $E_{b}=1.037-1.462\times planarity-0.068\times polarity$ with R^2^ = 0.934, and $E_{ex}=-0.460+0.538\times dipolemoment-0.440\times polarity$ with R^2^ = 0.965. These results highlight that solvent binding to the layer surface strengthens with increased planarity and reduced polarity, leading to a more negative $E_{b}$, which indicates better stabilization of the exfoliated layers. Conversely, the exfoliation energy $E_{ex}$ decreases with a higher dipole moment and lower polarity, signifying improved exfoliation efficiency. A high polarity and a large planarity are conducive to achieving a more negative binding energy, whereas a large dipole moment and a low polarity are beneficial to rendering a less negative exfoliation energy, implicating favorable exfoliation efficiency. Interestingly, polarity exhibits opposing effects on stabilization and exfoliation, requiring a balance during solvent selection. This trade-off positions dipole moment and planarity as crucial parameters for screening solvents. Among the solvents evaluated, dimethyl sulfoxide (DMSO) emerges as the optimal choice due to its high dipole moment and competitive planarity, which collectively enhance both the stabilization and exfoliation of Mg(OH)_2_ nanosheets. It is reported that the exfoliation efficacy of a solvent is enhanced when either the molecular planarity “sharpens” this molecular wedge or a strong phosphorene–solvent adhesion stabilizes the newly exposed phosphorene surface [14]. The molecular planarity in solvents like DMF enhances the cohesive energy [14].

### 3.5. Biaxial Straining Theory

The recently developed biaxial straining theory by Wu et al. [32,33,34,35,36] provides a framework for quantifying the atomistic fracture mechanics involved in nanomaterial exfoliation. The two forces applied along the two perpendicular axes yield biaxial straining to bring about the exfoliation of layered structures. This theory proposes that the combined contribution from multiple interactions, including electron–electron interactions governed by Coulomb’s law, electron–phonon interactions arising from lattice distortions, and phonon–phonon interactions caused by lattice vibrations, drives bond breaking, plastic deformation, and the exfoliation of layered materials. While simulating the liquid-phase exfoliation (LPE) process, it can be assumed that the binding of solvent molecules to the nanosheet’s surface and their insertion into layered structures generates the stress along the x and y directions. Consequently, the calculated binding energy shows a positive correlation with strain in the x direction. In contrast, the calculated exfoliation energy exhibits a negative correlation with strain in the y direction. The combined biaxial straining leads to the simulated Mg(OH)_2_ exfoliation. By simplifying the surface of Mg(OH)_2_ as a rectangle, mathematical derivation shows that the biaxial straining along the diagonal direction is maximized when the solvent generates equal strain in both the x and y directions. To quantify the difference between the two energy contributions, we define the biaxial straining index (ΔS) with weighted factors, as these contributions may vary in their impact on straining in the respective directions:

(1) $$∆S=A\cdot E_{b}-B\cdot E_{ex},$$

where A and B are weight factors for binding energy ($E_{b}$) and exfoliation energy ($E_{ex}$) in relation to the overall strain, respectively. In this study, A and B were standardized to 1 because their exact values are unknown, and our focus was on observing trends rather than determining specific ΔS values.

**Figure 5:**
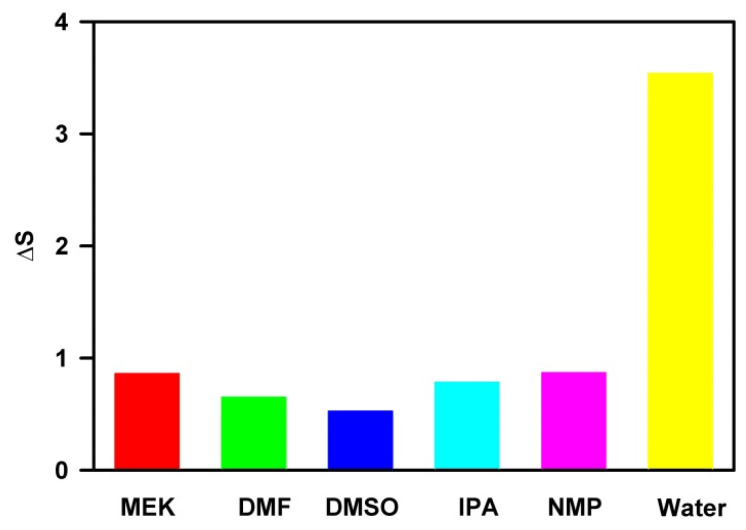
Calculated strain indices of the studied solvents.

Figure 5 displays the calculated strain index values for the solvents examined. A lower ΔS value is associated with greater biaxial straining along the diagonal direction, which, in turn, facilitates exfoliation. Among the solvents tested, DMSO exhibits the strongest biaxial straining effect, making it the most effective solvent for the liquid-phase exfoliation (LPE) of Mg(OH)_2_. In contrast, water has the highest biaxial straining index, suggesting that the biaxial straining it generates is the weakest among the solvents studied. As a result, water will be the least effective solvent for the LPE of Mg(OH)_2_, a finding that aligns with experimental observations on other layered materials [5,8,11,13].

### 3.6. Experimental Validation

**Figure 6:**
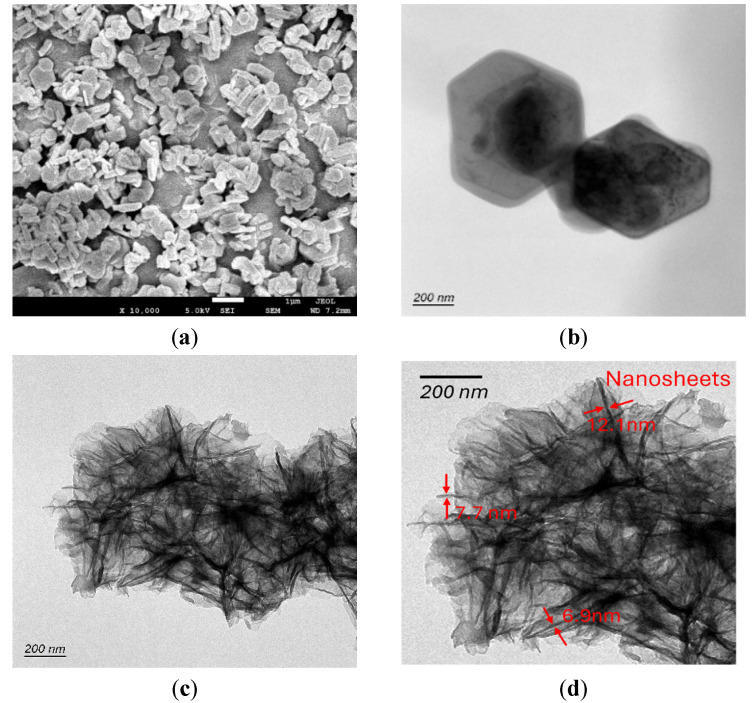
(**a**) SEM image of the bulk Mg(OH)_2_ particles; (**b**) TEM image of Sample A; (**c**) TEM image of Sample B; (**d**) Thickness of the nanosheets of sample B.

Experimentally, we employed solvent water and DMSO for the LPE of Mg(OH)_2_. Figure 6 shows the SEM and TEM images of bulk Mg(OH)_2_ powder and liquid-exfoliated Mg(OH)_2_ nanoparticles. As shown in Figure 6b, the TEM images of Sample-A exhibit hexagonal-shaped particles similar to those of the Mg(OH)_2_ bulk powder (Figure 6a), indicating an incomplete exfoliation process. In contrast, Figure 6c displays the TEM images of Sample B, exhibiting sheet-like structures, which indicates successful exfoliation into nanosheets. The average thickness of these nanosheets was measured at 8.1 nm (Figure 6d). Therefore, it is evident that the liquid exfoliation process employing DMSO generates nanosheets effectively. Comparatively, DMSO proves to be a more efficient solvent for generating Mg(OH)_2_ nanosheets compared to water as the liquid exfoliation medium.

## 4. Conclusions

Establishing quantitative criteria for solvent selection in liquid-phase exfoliation is essential for optimizing efficiency and performance. In this study, the exfoliation process was simulated in the following two distinct processes: (1) solvent intercalation—the energy required to insert a solvent molecule into the interlayer structure, reflecting the exfoliation energy, and (2) surface stabilization—the binding energy between the solvent molecule and the exfoliated surface, indicating the stabilization capacity. NMP exhibits the strongest interaction with the Mg(OH)_2_ surface, whereas MEK and IPA demonstrate the weakest interactions. On the other hand, DMSO demonstrates the greatest efficiency in weakening the interlayer attractions.

Our QSAR analysis revealed that binding energy increases with high polarity and large planarity of the solvent molecule, thus promoting surface stabilization, and the exfoliation energy decreases with high dipole moment and low polarity, enhancing the exfoliation efficiency. However, the contradictory effects of polarity on stabilization and exfoliation highlight the need for a balance in solvent properties. The biaxial straining theory proposes that minimizing the strain index, determined through weighted binding energy and exfoliation energy, leads to maximized biaxial strain, thereby facilitating exfoliation. Solvents with a high dipole moment and large planarity, such as DMSO, emerge as optimal candidates. Experimental observations confirmed that the liquid exfoliation process using DMSO produced nanosheets more effectively than using water. These findings establish quantitative criteria for solvent selection, guiding the exfoliation and stabilization of layered materials efficiently.

## Figures and Tables

**Table 1:** Selected properties of the solvents used in this work.

Name	Formula	Structure *	γ, mN/m [28]	Polarity [29]	Dipole Moment, D [29]	Planarity
water	H_2_O	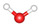	72.80	10.2	1.87	0.54
MEK	CH_3_C(O)CH_2_CH_3_	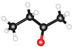	24.60	4.7	2.76	0.77
DMF	(CH_3_)_2_NC(O)H	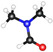	37.10	6.4	3.86	0.76
DMSO	(CH_3_)_2_SO	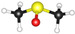	43.53	7.2	4.10	0.77
IPA	CH_3_CHOHCH_3_	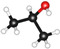	23.00	3.9	1.66	0.76
NMP	CH_3_N(CH_2_CH_2_CH_2_)C(O)	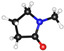	40.79	6.7	4.09	0.81

* Black, red, blue, yellow, and white balls represent C, O, N, S, and H atoms, respectively.

**Table 2:** Calculated binding energy $E_{b}$, exfoliation energy $E_{ex}$, and interlayer spacing $D_{i}$.

Solvent	$E_{b}$, eV	$E_{ex}$, eV	$D_{i}$, Å
− *		−5.55	2.50
H_2_O	−0.44	−3.98	2.77
MEK	−0.36	−1.22	6.03
DMF	−0.52	−1.17	6.08
DMSO	−0.58	−1.10	6.26
IPA	−0.37	−1.15	6.13
NMP	−0.60	−1.47	6.16

* indicates absence of solvent in the system.

## Data Availability

The data presented in this study are available upon request from the corresponding author.

## Abbreviations

The following abbreviations are used in this manuscript:

LPE	liquid-phase exfoliation
2D	two-dimensional
CVD	chemical vapor deposition
PVD	physical vapor deposition
MEK	2-butanone
DMF	dimethylformamide
DMSO	dimethyl sulfoxide
IPA	isopropyl alcohol
NMP	N-methyl-2-pyrrolidone
o-DCB	ortho-dichlorobenzene
HSP	Hansen solubility parameters
DFT	density functional theory
VASP	Vienna ab initio simulation package
PBE	Perdew–Burke–Ernzerhof
GGA	generalized gradient approximation
PAW	projector augmented wave
SEM	scanning electron microscopy
TEM	transmission electron microscopy
EDS	energy dispersive X-ray spectroscopy
PCA	principal component analysis
QSPR	quantitative structure–property relationship

## References

[1] Murali A., Lokhande G., Deo K.A., Brokesh A., Gaharwar A.K. (2021). Emerging 2D nanomaterials for biomedical applications. Mater. Today.

[2] Noreen S., Tahir M.B., Hussain A., Nawaz T., Rehman J.U., Dahshan A., Alzaid M., Alrobei H. (2022). Emerging 2D-nanostructured materials for electrochemical and sensing application—A review. Int. J. Hydrog. Energy.

[3] Alam S., Asaduzzaman Chowdhury M., Shahid A., Alam R., Rahim A. (2021). Synthesis of emerging two-dimensional (2D) materials—Advances, challenges and prospects. FlatChem.

[4] Baig N. (2023). Two-dimensional nanomaterials: A critical review of recent progress, properties, applications, and future directions. Compos. Part A-Appl. Sci. Manuf..

[5] Kaur H., Coleman J.N. (2022). Liquid-Phase Exfoliation of Nonlayered Non-Van-Der-Waals Crystals into Nanoplatelets. Adv. Mater..

[6] Xu Y.Y., Cao H.Z., Xue Y.Q., Li B., Cai W.H. (2018). Liquid-Phase Exfoliation of Graphene: An Overview on Exfoliation Media, Techniques, and Challenges. Nanomaterials.

[7] Telkhozhayeva M., Teblum E., Konar R., Girshevitz O., Perelshtein I., Aviv H., Tischler Y.R., Nessim G.D. (2021). Higher ultrasonic frequency liquid phase exfoliation leads to larger and monolayer to few-layer flakes of 2D layered materials. Langmuir.

[8] Hernandez Y., Nicolosi V., Lotya M., Blighe F.M., Sun Z.Y., De S., McGovern I.T., Holland B., Byrne M., Gun’ko Y.K. (2008). High-yield production of graphene by liquid-phase exfoliation of graphite. Nat. Nanotechnol..

[9] Huo C.X., Yan Z., Song X.F., Zeng H.B. (2015). 2D materials via liquid exfoliation: A review on fabrication and applications. Sci. Bull..

[10] Li Z.L., Young R.J., Backes C., Zhao W., Zhang X., Zhukov A.A., Tillotson E., Conlan A.P., Ding F., Haigh S.J. (2020). Mechanisms of liquid-phase exfoliation for the production of graphene. ACS Nano.

[11] Hernandez Y., Lotya M., Rickard D., Bergin S.D., Coleman J.N. (2010). Measurement of multicomponent solubility parameters for graphene facilitates solvent discovery. Langmuir.

[12] Fernandes J., Nemala S.S., De Bellis G., Capasso A. (2022). Green solvents for the liquid phase exfoliation production of graphene: The promising case of cyrene. Front. Chem..

[13] Manna K., Huang H.N., Li W.T., Ho Y.H., Chiang W.H. (2016). Toward Understanding the efficient exfoliation of layered materials by water-assisted cosolvent liquid-phase exfoliation. Chem. Mater..

[14] Sreshtt V., Pádua A.A.H., Blankschtein D. (2015). Liquid-phase exfoliation of phosphorene: Design rules from molecular dynamics simulations. ACS Nano.

[15] Suslu A., Wu K., Sahin H., Chen B., Yang S., Cai H., Aoki T., Horzum S., Kang J., Peeters F.M. (2016). Unusual dimensionality effects and surface charge density in 2D Mg(OH)_2_. Sci. Rep..

[16] Kresse G., Furthmuller J. (1996). Efficient iterative schemes for ab initio total-energy calculations using a plane-wave basis set. Phys. Rev. B.

[17] Hammer B., Hansen L.B., Norskov J.K. (1999). Improved adsorption energetics within density-functional theory using revised Perdew-Burke-Ernzerhof functionals. Phys. Rev. B.

[18] Kresse G., Joubert D. (1999). From ultrasoft pseudopotentials to the projector augmented-wave method. Phys. Rev. B.

[19] Kresse G., Furthmuller J. (1996). Efficiency of ab-initio total energy calculations for metals and semiconductors using a plane-wave basis set. Comput. Mater. Sci..

[20] Grimme S., Antony J., Ehrlich S., Krieg H. (2010). A consistent and accurate ab initio parametrization of density functional dispersion correction (DFT-D) for the 94 elements H-Pu. J. Chem. Phys..

[21] Monkhorst H.J., Pack J.D. (1976). Special points for brillouin-zone integrations. Phys. Rev. B.

[22] BIOVIA, Dassault Systèmes (2014). Material Studio.

[23] Eisenhaber F., Argos P. (1993). Improved strategy in analytic surface calculation for molecular systems: Handling of singularities and computational efficiency. Comput. Chem..

[24] Connolly M.L. (1983). Solvent-accessible surfaces of proteins and nucleic acids. Science.

[25] Wu S., Senevirathna H.L., Weerasinghe P.V.T., Wu P. (2021). Engineering electronic structure and band alignment of 2D Mg(OH)_2_ via anion doping for photocatalytic applications. Materials.

[26] Zhang D.Y., Zhang P.X., Song S.H., Yuan Q.H., Yang P., Ren X.Z. (2014). Simulation of magnesium hydroxide surface and interface. J. Alloys Compd..

[27] Momma K., Izumi F. (2011). VESTA 3 for three-dimensional visualization of crystal, volumetric and morphology data. J. Appl. Crystallogr..

[28] Lechner M.D. (1997). Surface Tension of Pure Liquids and Binary Liquid Mixtures.

[29] Bonin K.D., Kresin V.V. (1997). Electric-Dipole Polarizabilities of Atoms, Molecules, and Clusters.

[30] Wu S.N., Wu P. (2023). Energetic and configurational mechanisms to facilitate mica nanosheets synthesis by organo-ammonium cation intercalation. Comput. Mater. Sci..

[31] Jeffrey G.A. (1997). An Introduction to Hydrogen Bonding.

[32] Wu P., Thenuwara H.N., Senevirathna H.L. (2023). Entropy-driven liquid-phase exfoliation of non-Van-Der-Waals crystals into nanoplatelets. FlatChem.

[33] Tan B.T., Wu P., Anariba F. (2022). Modeling stress-strain nonlinear mechanics via entropy changes on surface wetting using the Born-Oppenheimer approximation. Results Eng..

[34] Weerasinghe P.V.T., Wu S., Lee W.P.C., Zhu Q., Lin M., Wu P. (2024). Mica nanosheets synthesized via liquid Ga embrittlement: Demonstrating enhanced CO_2_ capture. Mater. Adv..

[35] Lee W.P.C., Wu S., Anariba F., Wu P. (2023). Breaking new ground in mica exfoliation: Harnessing biaxial straining principles through H_2_ and N_2_ intercalation for enhanced layer separation. Mater. Today Adv..

[36] Wu S., Weerasinghe P.V.T., Wu P. (2023). Fostering mica exfoliation through biaxial straining strategy with monovalent cation substitution. FlatChem.

